# Gene therapy strategies for aging intervention

**DOI:** 10.1016/j.cellin.2025.100254

**Published:** 2025-05-23

**Authors:** Yaobin Jing, Jie Ren, Jing Qu, Guang-Hui Liu

**Affiliations:** a International Center for Aging and Cancer, Hainan Academy of Medical Sciences, Hainan Medical University, Haikou 571199, Hainan, China; b State Key Laboratory of Organ Regeneration and Reconstruction, Institute of Zoology, Chinese Academy of Sciences, Beijing 100101, China; c Beijing Institute for Stem Cell and Regenerative Medicine, Beijing 100101, China; d Aging Biomarker Consortium, Beijing 100101, China; e University of Chinese Academy of Sciences, Beijing 100049, China; f Key Laboratory of RNA Innovation, Science and Engineering, China National Center for Bioinformation, Beijing 100101, China; g Beijing Institute of Genomics, Chinese Academy of Sciences, Beijing 100101, China; h Sino-Danish College, University of Chinese Academy of Sciences, Beijing 101408, China; i School of Future Technology, University of Chinese Academy of Sciences, Beijing 100049, China

## Abstract

Aging is characterized by a progressive decline in organ and tissue structure and function, significantly increasing the risk of many chronic diseases. Developing interventions to delay aging holds the potential to reduce the burden of age-associated diseases and promote healthy longevity. Gene therapy has emerged as a clinically transformable approach, leveraging advanced gene editing and delivery systems to target the molecular underpinnings of aging. This review systematically explores the potential of gene therapy strategies in aging intervention, focusing on approaches that enhance genomic and epigenetic stability, restore metabolic homeostasis, modulate immune responses, and rejuvenate senescent cells. By providing a comprehensive overview and forward-looking insights, this article aims to inform future research directions and translational applications of gene therapy in mitigating aging-related decline.

## 1 Introduction

Aging is a multifaceted process involving progressive decline in physiological functions, with pronounced features encompassing weakened tissue repair and regeneration abilities, declined metabolic capability, dysfunctional immune system, and increased sterile inflammation, leading to markedly increased susceptibility to various degenerative diseases, such as cardiovascular and neurodegenerative disorders (Borghesan et al., 2020; Cai et al., 2022; López-Otín et al., 2013). This decline is driven by a set of underlying molecular and cellular changes, such as genomic instability, telomere attrition, epigenetic dysregulation, proteostasis imbalance, and mitochondrial dysfunction (Liu et al., 2022; Bao et al., 2023; López-Otín et al., 2023). These changes are interconnected and collectively contribute to the aging process, making them critical targets for interventions aimed at extending healthspan and lifespan.

A deeper understanding of aging-related changes and their underlying mechanisms has propelled the development of a diverse array of aging intervention strategies. Among these, small molecule therapeutics, such as metformin and ascorbic acid, lithocholic acid, taurine, uridine, have shown beneficial effects in modulating aging markers and promoting functional recovery (Geng et al., 2023; Liu, Li, et al., 2022; Qu et al., 2024; Singh et al., 2023; Sun et al., 2023; Yang et al., 2024). Recently, the use of senolytic agents has emerged as a promising approach to directly eliminate senescent cells and alleviate age-related symptoms. For example, the combination of dasatinib and quercetin (D ​+ ​Q) effectively reduces senescent cell burden, restoring vitality in aged mice (Chaib et al., 2022; Novais et al., 2021). Despite these advances, challenges remain in achieving precise targeting, long term efficacy, and translating findings from model organisms to humans. Additionally, the overall timeline from target discovery, small molecule screening, and drug evaluation to clinical application is relatively long, which significantly constrains the development and widespread application of small molecule drugs (Gonçalves & Paiva, 2017; Sayed et al., 2022).

In contrast, gene therapy technology offers transformative potential by enabling precise genetic modifications and targeted delivery to aged tissues. Advances in gene editing tools, represented by clustered regularly interspaced short palindromic repeats (CRISPR)-Cas9 (Doudna & Charpentier, 2014), base editing (Komor et al., 2016), and prime editing (Scholefield & Harrison, 2021), have revolutionized the modulation of genetic and epigenetic factors associated with aging. Concurrently, optimized delivery systems, including adeno-associated virus (AAV) and lipid nanoparticles (LNPs), enhance targeting efficiency (Manikandan et al., 2020; Yin et al., 2014). These advances offer innovative and robust approaches for targeting and modulating aging-related regulatory pathways, promoting a transformative shift in aging intervention from "symptom relief" to "mechanism addressing", while simultaneously accelerating the research and development process.

This review highlights the most recent progress in gene therapy strategies for aging intervention, with a particular emphasis on innovative gene editing approaches and their translational potential. We discuss representative gene-targeted interventions, elucidating their mechanisms and therapeutic prospects, and critically assess challenges and future perspectives in achieving safe and effective clinical applications.

## 2 The development of gene therapy

1. **Gene therapy strategies**
  Gene therapy provides revolutionary solutions for genetic disorders, cancer, and degenerative diseases, etc., through precise correction of pathogenic gene mutations or regulation of key biological pathways. From a gene expression regulation standpoint, gene therapy primarily employs four strategies (Fig. 1(A)).
  ① Gene editing: Utilizing technologies such as CRISPR-Cas9, base editing, and prime editing to precisely edit the genome or achieve specific gene expression regulation (Li et al., 2023c; Scholefield & Harrison, 2021; Wu, Wan, et al., 2023). For example, correct the mutation site in the *HBB* gene using base editing technology to treat sickle cell disease (Desai et al., 2024). These technologies offer unprecedented potential for aging intervention but also pose challenges related to off-target effects and genomic stability, requiring rigorous safety evaluation (Pacesa et al., 2024).
  ② Transcriptional regulation: Incorporating catalytically dead Cas9 (dCas9) with transcription repressor (i.e., KRAB), activators (i.e., VP64, p65) or epigenetic modifications enzymes (i.e., P300, DNMT3A) to regulate gene expression (Hilton et al., 2015; Konermann et al., 2015; Larson et al., 2013; Park et al., 2022). Relative to genome editing, this approach facilitates dynamic and reversible regulation of gene expression, providing therapeutic potential in conditions where gene expression modulation is required without permanent alterations (Liu et al., 2025).
  ③ Gene replacement and overexpression: Delivering functional genes to compensate for defective or lost gene or to upregulate gene expression. A prime example is the gene therapy drug Luxturna, which delivers normal copies of the *RPE65* gene to retinal cells, improving vision in patients with RPE65 deficiency (Leroy et al., 2023). While successful in some cases, challenges remain regarding immunogenicity and long-term stability of gene expression (Magnani et al., 2022).
  ④ Gene silencing: inhibiting gene expression based on small interfering RNA (siRNA), antisense oligonucleotides (ASO), and other RNA interference technologies. For example, the ASO drug Wainua treats amyloidosis polyneuropathy by suppressing transthyretin (TTR) production (Coelho et al., 2021). Gene silencing strategies are valuable for targeting pathogenic gene expression but may face challenges related to delivery and duration of effect (Prakash et al., 2024).

**Fig. 1:**
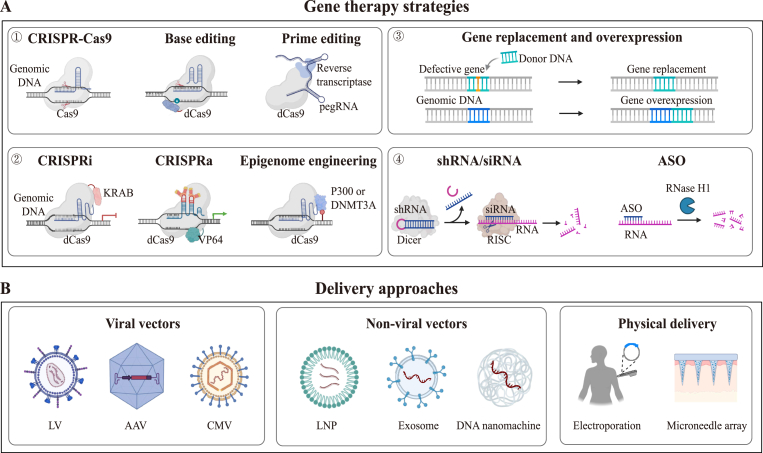
**Gene therapystrategiesand deliveryapproaches.** **(A**) Gene therapy strategies including ① Gene knockout or correction with CRISPR-Cas9, base editing or prime editing. ② Gene regulation mediated with CRISPRi (fusing dCas9 with KRAB), CRISRPa (fusing dCas9 with VP64-p65) and epigenome engineering (fusing dCas9 with P300 or DNMT3A). ③ Introduction of functional genes to compensate gene defects or upregulate gene expression. ④ Gene silencing with shRNA/siRNA or ASO. (**B**) Delivery systems for gene therapy including viral vectors (LV, AAV, CMV), non-viral vectors (LNP, exosome, DNA nanomachine) and physical methods (electroporation and microneedle array). CRISPRi, CRISPR inhibition. CRISPRa, CRISPR activation. shRNA, short hairpin RNA. siRNA, small interference RNA. RISC, RNA-induced silencing complex. ASO, antisense oligonucleotides. LV, lentivirus. AAV, adeno-associated virus. CMV, cytomegalovirus. LNP, lipid nanoparticle.
2. **Gene delivery approaches**

Concurrently, innovations in gene delivery approaches have notably enhanced targeting efficacy and safety (Fig. 1(B)–Table 1). Viral vectors, particularly AAV, are widely used due to their non-integrative nature and low immunogenicity, making them suitable for long-term treatment, thus are FDA-approved gene therapy vectors (Au et al., 2021; Wang, Gessler, et al., 2024). More importantly, through serotype optimization, AAV vectors can achieve efficient delivery to specific organs. For example, AAV-BI30, developed based on AAV9, can penetrate the blood-brain barrier to target brain endothelial cells (Krolak et al., 2022); AAV-ie vectors with cell-penetrating peptides specifically target cochlear supporting cells and hair cells (Tan et al., 2019). These optimized AAVs enhance therapeutic effects while reducing potential impacts on non-target organs. Despite their advantages, the packaging capacity of AAVs is limited to approximately 5 kb, which restricts the size of the therapeutic gene that can be delivered. Additionally, pre-existing neutralizing antibodies in the human population might limit the efficacy of AAV-based therapies (Mendell et al., 2022). Furthermore, AAV vectors may trigger immune responses, warranting careful consideration of safety (Ronzitti et al., 2020).

**Table 1:** Delivery approaches for gene therapy.

Delivery system	Cargo capacity	Tropism	Integration	Expression Durability	Immunogenicity	Citation
AAV	∼5 kb	Serotype-dependent	Non-integrating	Long-term (months-years)	Low-moderate	Benatti et al. (2024)
LV	8–10 kb	Broad (promoter-dependent)	Integrating	Long-term (integration)	High	Bulcha et al. (2021)
LNP	∼10 kb	Liver-predominant	Non-integrating	Short-term (weeks)	Moderate-high	(Xu et al., 2025, Hosseini-Kharat et al., 2025)
Exosome	∼5 kb	Native tropism	Non-integrating	Short-term	Very low	(Balaraman et al., 2025; Ahmadi et al., 2023)

Compared to AAV, lentivirus (LV) possess important applications in gene therapy and cell therapy due to their efficient and stable genome integration, robust gene-carrying capacity (8–10 kb), and broad cell infection capabilities, showing unique advantages particularly in *ex vivo* modification of specific cell types such as CAR-T therapy (Bulcha et al., 2021; Raguram et al., 2022; Zhang et al., 2025). By replacing heterologous surface glycoproteins, the binding ability of LV to target cells can be further enhanced, thereby improving transduction efficiency. For instance, VSV-G pseudotyped LV is widely applied in mammalian cell transduction and has reconstructed immune function in X-SCID patients in clinical trials (Wolff & Mikkelsen, 2022). However, the integration of LV into the host genome increases the risk of insertional mutagenesis and oncogenesis (Hacein-Bey-Abina et al., 2008). LV also have higher immunogenicity compared to AAVs (Chen et al., 2020). Hence, the need for careful vector design and selection remain significant challenges.

Additionally, gene delivery approaches have also achieved breakthrough in non-viral vectors, including LNPs, exosome and DNA nanomachines. LNPs efficiently encapsulate Cas9 mRNA and sgRNA, overcoming the low delivery efficiency of traditional non-viral vectors (Piao et al., 2025; Ma et al., 2024a). LNPs can be engineered with targeting moieties, such as ligands, signal peptides, or antibodies to achieve cell- or tissue-specific delivery. This targeted approach enables LNPs to deliver therapeutic agents to specific sites, such as the liver or immune cells. While LNPs offer advantages in terms of versatility and flexibility, their lipid composition presents potential toxicity concerns. Specifically, ionizable lipids may interact with Toll-like receptors (TLRs), potentially triggering inflammatory responses (Chaudhary et al., 2024). Furthermore, controlling the stability and biodistribution of LNPs remains challenging, and their therapeutic efficacy can vary considerably depending on both the formulation and target tissue (Ma, Li, et al., 2024; Zhang & Barz, 2025).

Exosomes are naturally occurring extracellular vesicles (30–150 nm) that can be utilized as efficient delivery vehicles for genetic material, including mRNA and CRISPR components. These vesicles possess several therapeutic advantages: they exhibit inherent low immunogenicity, can be engineered for tissue-specific targeting through surface protein modification, and naturally protect their cargo from enzymatic degradation while evading neutralizing antibodies (Kim et al., 2024, Li et al., 2025). Their tropism can be exploited therapeutically—for instance, exosomes derived from neural stem cells (NSCs) demonstrate preferential accumulation in brain (Webb et al., 2018). This tissue-specific homing, combined with their ability to cross biological barriers, positions exosomes as a promising approach for delivering therapeutic agents to challenging target tissues such as the brain or muscle, particularly for addressing aging-related pathologies (Liu et al., 2024). Despite these advantages, significant technical challenges persist. Large-scale production and purification of therapeutically viable exosomes remain complicated and costly. Consequently, developing scalable manufacturing methods and standardized purification protocols represents a critical focus of current research efforts. Interestingly, DNA nanomachines, through structural optimization to improve biocompatibility, demonstrate advantages in controlled loading and intelligent delivery of biomacromolecules (Ramezani & Dietz, 2020).

Meanwhile, physical delivery methods (such as electroporation and microneedle arrays) further expand gene therapy delivery systems, providing new approaches for gene therapy in specific tissues or cells, such as gene editing for skin and immune cells. These novel delivery approaches efficiently transport therapeutic gene editing tools to target cells, thereby enhancing therapeutic effects while reducing side effects (Raguram et al., 2022). They have laid the foundation for the widespread application of gene therapy and driven the field toward more precise and safer development.

## 3 Gene therapy for aging interventions

**Fig. 2:**
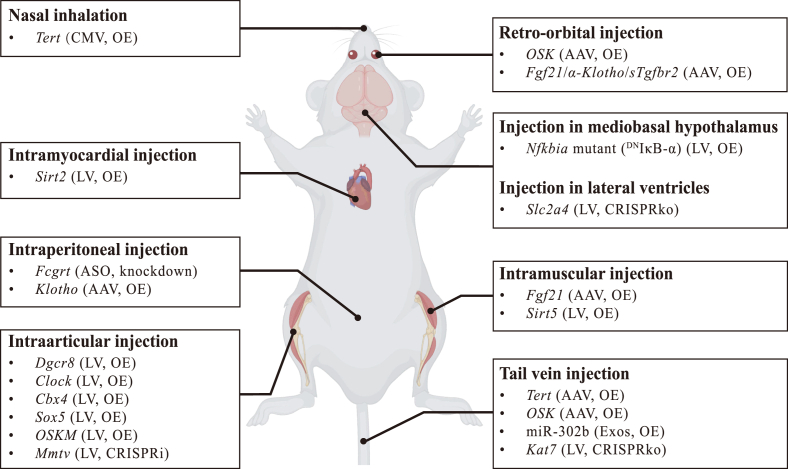
***In vivo*****gene therapy delivery strategies and targets for aging intervention.** Gene therapy vectors can be delivered *in vivo* through various methods, including nasal inhalation (*Tert* overexpression with CMV), retro-orbital injection (*OSK* or *Fgf21*/*α-Klotho*/*sTgfbr2* overexpression with AAV), tail vein injection (*Tert* or OSK overexpression with AAV, miR-302b overexpression with Exos, CRISPRko of *Kat7* with LV), intraperitoneal injection (*Fcgrt* knockdown with ASO, *Klotho* overexpression with AAV), intra-cerebroventricular (mediobasal hypothalamus, lateral ventricles) injection (*Nfkbia* mutant (^DN^IκB-α) overexpression with LV, CRISPRko of *Slc2a4* with LV), intramuscular injection (*Fgf21* overexpression with AAV, *Sirt5* overexpression with LV), intramyocardial injection (*Sirt2* overexpression with LV) and intraarticular injection (*Dgcr8*, *Clock*, *Cbx4*, *Sox5*, *OSKM* overexpression with LV, CRISPRi of *Mmtv* with LV). OE, overexpression. CMV, cytomegalovirus. LV, lentivirus. AAV, adeno-associated virus. Exos, exosomes. CRISPRko, CRISPR-mediated knockout. CRISPRi, CRISPR-mediated inhibition. *OSKM*, *Oct4*/*Sox2*/*Klf4*/*c-Myc*.

**Fig. 3:**
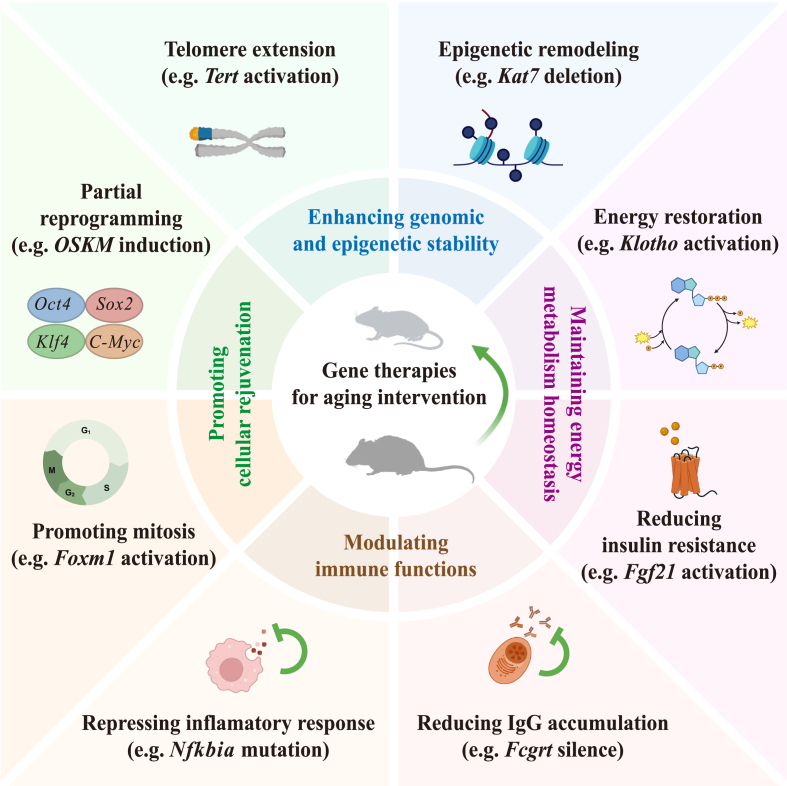
**Gene therapy strategies for delaying aging.** The schematic diagram illustrates gene therapy for aging intervention in mice through mechanisms including: enhancing genomic and epigenetic stability by telomere extension (*Tert* activation) and epigenomic remodeling (*Kat7* deletion); maintaining energy metabolism homeostasis by energy restoration (*Klotho* activation) and reduction of insulin resistance (*Fgf21* activation); modulating immune functions by repressing inflammatory response (*Nfkbia* mutation) and reducing IgG accumulation (*Fcgrt* silence); and promoting cellular rejuvenation by partial reprogramming (*OSKM* induction) and promoting mitosis (*Foxm1* activation).

The development of gene therapy technologies has provided new avenues for aging intervention, demonstrating unique advantages. Compared to traditional approaches such as drug treatments and lifestyle interventions, gene therapy utilizes delivery vectors to achieve *in vivo* inhibition or activation of key regulatory genes or pathways involved in aging, offering greater potential for delaying aging and extending healthy lifespan (Liu & Izpisua Belmonte, 2022; Cai et al., 2022; Bernardes de Jesus et al., 2012; Wang, Zheng, et al., 2021). Here, we systematically elaborate on current research progress in gene therapy for aging intervention from aspects of enhancing genomic and epigenetic stability, maintaining energy metabolism homeostasis, modulating immune functions, and promoting cellular rejuvenation (Fig. 2, Fig. 3). Through this comprehensive review, we aim to elucidate the future developmental trajectory and translational potential of gene therapy approaches in aging intervention.

(1). **Gene therapy for genome stabilization and epigenetic remodeling**

The progressive loss of genomic stability and imbalance in epigenetic homeostasis constitute one of the key characteristics of aging. Manifestations of genomic instability include accumulation of DNA damage and gene mutations, telomere shortening, and chromosomal abnormalities, primarily caused by decreased DNA damage repair capacity, abnormal function of telomere protein complexes, telomerase inactivation, and abnormal chromosomal recombination (López-Gil et al., 2023; Venkatesan et al., 2015). Telomeres, DNA-protein complexes at the ends of eukaryotic chromosomes, maintain genomic integrity by capping chromosome ends. However, with cell division, telomere DNA length gradually shortens, eventually causing chromatin to undergo fusion and breakage due to loss of telomere protection, triggering sustained DNA damage responses that lead to cell cycle arrest and cellular senescence (Lu & Liu, 2024; Wu, Qu, & Liu, 2024). Therefore, telomere extension is considered a potential means of delaying aging.

Telomerase is a ribonucleoprotein complex that maintains telomere length, composed of telomerase reverse transcriptase (TERT) and telomerase RNA (TR). TERT activity gradually decreases during the aging process, and activating telomerase or TERT helps delay aging. Bernardes de Jesus et al. and Jaijyan et al. delivered *TERT*-expressing vectors to aged mice via AAV9 and CMV, extending telomere length in multiple tissues and organs, effectively improving the structural and functional regression of aging organs, including restored hair density, increased bone density and muscle mass (Bernardes de Jesus et al., 2012; Jaijyan et al., 2022). However, it should be noted that telomerase is typically expressed at low levels in normal somatic cells, and its continuous activation may increase the risk of carcinogenesis by promoting abnormal cell proliferation (Florez-Vargas et al., 2025; Iskandar et al., 2025; Shim et al., 2024). Therefore, precise control of intervention timing and dosage is crucial.

Gene mutations accumulate during the aging process. Through comprehensive genomic analysis of nine human organs, Li et al., revealed that somatic mutation accumulation in normal tissues is closely associated with aging and the occurrence of age-related diseases (Li et al., 2021). Additionally, DNA repair defects accelerate human aging, as evidenced by patients with RecQ helicase genetic mutations who exhibit characteristics of premature aging, suggesting a correlation between DNA damage repair capacity and organismal aging (Zhang et al., 2015). Studies have demonstrated that promoting DNA damage repair contributes to maintaining genomic stability and delaying cellular senescence. For instance, YIPF2 promotes BRCA1 and Rad51-mediated homologous recombination repair, retarding DNA damage-induced cellular senescence, indicating that targeted expression of specific genes related to DNA damage repair has potential for aging intervention (Zhang & Wang, 2024). Currently, there is no conclusive research evidence demonstrating that enhancing DNA repair capacity through gene therapy approaches extends lifespan. This may be due to the complex regulatory mechanisms involving multiple enzymatic pathways in DNA damage repair processes, as well as the need for optimization of therapeutic strategies, including precise control of treatment windows, appropriate dose adjustments, and accurate selection of targeted tissues, organs, or cell types (MacRae et al., 2015; Petr et al., 2020; Schumacher et al., 2021; Chen et al., 2024b). Therefore, in-depth analysis and identification of DNA damage repair factors with aging intervention capabilities represent an important direction warranting further investigation.

Multiple epigenetic dimensions, including DNA methylation, histone modifications, RNA modifications, and non-coding RNA expression, participate in the aging process (Ren et al., 2017; Wu, Zhang, et al., 2024). On one hand, epigenetics is closely related to genomic stability and nuclear lamina disintegration, together leading to heterochromatin disassembly, such as the overall loss of histone modification H3K9me3 and DNA methylation, which activates transposition of repetitive sequences (e.g., LINE-1 transposable elements) (Bi et al., 2024; De Cecco et al., 2019; Gorbunova et al., 2021; Liu, Ji, et al., 2022; Wang et al., 2022; Zhang et al., 2020). These elements subsequently spread throughout the genome via "transcription-reverse transcription-insertion" mechanisms and assist other transposons (such as SINEs) in transposition, increasing recombination probability between different genes, disrupting genomic stability, and ultimately triggering aging-related diseases (Li, Yu, et al., 2024b; Richardson et al., 2015). Researches in cellular models have found that maintaining heterochromatin levels and suppressing repetitive sequences effectively delay cellular senescence. Similarly, delivering heterochromatin maintenance factors (such as CLOCK, BMAL1, DGCR8, and CBX4) to mouse joint cavities using LV improved age-related cartilage degeneration, indicating that maintaining heterochromatin stability through gene therapy approaches is a potential strategy for delaying aging (Deng et al., 2019; Liang et al., 2021, 2022; Ren et al., 2019). Concurrently, reverse transcription products of repetitive sequences (including LTR/ERVs, LINE-1) significantly increase in senescent cells and tissues, triggering cGAS-STING pathway-mediated innate immune responses, leading to senescence-associated secretory phenotype (SASP) and accelerating the aging process of various organs (De Cecco et al., 2019; Liu et al., 2023; Zhang et al., 2023). CRISPRi systems targeting the regulatory elements of mouse mammary tumor virus (*Mmtv*), an active ERVs in mouse, effectively alleviated aging-related articular degeneration with structural and functional improvements of joints (Liu et al., 2023). Suppressing *Mmtv* transcription and reverse transcription levels using gene therapy approaches retarded neuron senescence, holding the potential to improve cognitive abilities and joint mobility (Zhang et al., 2023).

On the other hand, epigenetic changes cause transcriptional dysregulation that promotes the aging process. For instance, histone deacetylase KAT7 promotes expression of the cell cycle regulator p15^INK4b^ by catalyzing acetylation of H3K14 (H3K14ac), accelerating senescence (Wang, Zheng, et al., 2021). Wang et al. has found that targeted knockout of *Kat7* in aged mouse livers using LV reduces p15^INK4b^ level in hepatocytes, rejuvenates senescent cells in the liver, improves metabolic function, and effectively extends mouse lifespan (Wang, Zheng, et al., 2021). Similarly, a reduction in the expression levels of METTL3, the core methyltransferase for RNA m^6^A modification, leads to decreased mRNA m^6^A levels of the skeletal muscle homeostasis regulatory gene *NPNT*, thereby disrupting this protein's stability and accelerating skeletal muscle aging (Wu, Lu, et al., 2023). Additionally, non-coding RNAs, including lncRNAs, microRNAs, and circRNAs, regulate gene expression by modulating chromatin epigenetic states or targeting transcript splicing and inhibiting translation, thereby regulating the aging process. For example, Bi et al. discovered that miR-302b present in human embryonic stem cell exosomes directly targets the 3′UTR sequences of *Cdkn1a* and *Ccng2*, inhibiting gene transcription, thereby promoting cell proliferation and delaying cellular senescence. Delivering miR-302b to aged mice via exosomes restored age-related hair loss and memory decline, reduced inflammation levels in multiple tissues including kidneys, liver, and skin, and extended lifespan (Bi et al., 2025).

(2). **Gene therapy for maintaining energy metabolism homeostasis**

Organismal aging is accompanied by metabolic dysfunction and energy homeostasis imbalance. In recent years, based on deepened understanding of metabolic alterations during aging, researchers have developed approaches using gene therapy to regulate metabolism and subsequently intervene in aging (Huang et al., 2025; Sun et al., 2025; Wiley & Campisi, 2021). Fibroblast growth factor 21 (FGF21) is a hormone secreted by hepatocytes with functions including regulation of glucose and lipid metabolism, and resistance to oxidative stress (Bondurant & Potthoff, 2018; Yano et al., 2022). FGF21 shows positive effects on treating metabolism-related diseases such as atherosclerosis and cardiovascular diseases through reducing hepatic triglycerides, enhancing insulin sensitivity, and increasing energy production (Geng et al., 2020; Harrison et al., 2024; Tan et al., 2023). Jimenez et al. injected AAV1-FGF21 into aged mouse muscle, promoting long-term increased circulating levels of FGF21 in the blood, which reversed age-related insulin resistance, weight gain, and cognitive decline, extending the healthy lifespan of mice (Jimenez et al., 2024).

Klotho is a family of single-chain transmembrane proteins closely associated with human aging, including α-Klotho, β-Klotho, and γ-Klotho. Their primary function is to assist FGF family proteins in exerting metabolic regulatory effects (Kim et al., 2015; Kuro-O, 2019). α-Klotho is mainly expressed in kidneys and thyroid, regulating phosphate and vitamin D3 metabolism through FGF23. β-Klotho is primarily expressed in liver and adipose tissue, binding with FGF15/19 and FGF21 to regulate bile production and energy metabolism. The transmembrane form of Klotho undergoes hydrolysis to produce a secreted form of the protein, which participates in regulating bodily functions as a circulating hormone in serum (Buchanan et al., 2020; Harrison et al., 2024). Early research showed that Klotho-deficient mice exhibited accelerated aging phenotypes with short lifespan (Kuro-o et al., 1997). Roig-Soriano et al. used AAV9 to deliver two isoforms of α-Klotho to the cerebral ventricle of premature aging model (SAMP8), expressing both the transmembrane protein form and secreted form of Klotho proteins, respectively (Roig-Soriano et al., 2022). They found that both forms of α-Klotho improved mouse cognitive abilities and physical condition, alleviating age-related cortical bone thickening and neuroinflammation. Simultaneously, age-related molecular characteristics are improved, including deceleration of the DNA methylation clock and reduction of senescent cells and inflammation level in brain (Roig-Soriano et al., 2022).

Metabolic abnormalities in the elderly typically manifest as interactive effects of multiple abnormalities including obesity, insulin resistance, glucose abnormalities, dyslipidemia, and hypertension (Lechleitner, 2008; Wang et al., 2014). Gene therapy delivering multiple genes simultaneously provides a potential solution to this challenge. For example, Davidsohn et al. used AAV8 to deliver FGF21, α-Klotho, and soluble mouse transforming growth factor β receptor 2 (sTGFβR2) individually or in combination to mouse models of aging-related diseases, including obesity, type 2 diabetes, heart failure, and renal failure (Davidsohn et al., 2019). Results showed that both individual and combination therapies produced therapeutic effects for different diseases. Among them, the FGF21 and sTGFβR2 combination simultaneously alleviated symptoms of four aging-related diseases, suggesting the potential of combination gene therapy in simultaneously preventing and treating multiple age-related chronic diseases and improving health status.

(3). **Gene therapy for modulating inflammatory pathways**

Chronic inflammation is closely associated with and contributes to the development of multiple age-associated diseases (Zhang et al., 2023a; Sun, Yue, & Wang, 2024; Basisty et al., 2020; Birch & Gil, 2020; Wang, Han, et al., 2024). cGAS was activated by sensing cytosolic DNA to synthesize the second messenger cyclic GMP-AMP (cGAMP), which binds and activates STING followed by recruitment and phosphorylation of TBK1. As the downstream responses of cGAS-STING innate immune pathway, TBK1 activates both IRF3 and NF-κB to promote transcription of inflammatory cytokines. Targeting key nodes in cGAS-STING pathway through gene therapy to suppress inflammation provides new possibilities for delaying aging and enhancing health. Recently, Zhao et al. demonstrated that SIRT5-mediated desuccinylation of TBK1 facilitated it's dephosphorylation and suppressed the downstream pro-inflammatory response. Intramuscular injection of *Sirt5* using LV ameliorated skeletal muscle dysfunction in aged mice (Zhao et al., 2025). In addition, NF-κB is a key transcriptional regulator that orchestrates immune signaling cascades and proinflammatory cytokine production, with established mechanistic links to age-related activation of SASP (Adler et al., 2007). Zhang et al. used LV to deliver a dominant negative mutant of *Nfkbia* (encoding ^DN^IκB-α) to the mediobasal hypothalamus (MBH) of aged mice, inhibiting NF-κB nuclear translocation and thereby blocking NF-κB activation (Zhang et al., 2013). They found that aging-related characteristics such as muscle atrophy were reversed in mice, and lifespan was extended. However, cGAS knockout mice displayed premature aging phenotypes with increased expression of LINE-1 and dysregulated inflammatory activity. This implies additional roles of cGAS in gene expression regulation (Martinez et al., 2024). Hence gene therapies targeting cGAS pathway should be designed and implemented with caution.

Simultaneously, immunoglobulin G (IgG), the main type of circulating antibody in serum, plays an important role in resisting viral and bacterial invasions and in anti-infection. Ma et al. discovered a high correlation between tissue aging and accumulation of humoral immune reaction-related genes such as *IgKC* (Ma et al., 2024a). The accumulation of IgG promotes the senescence of macrophages and microglia, and induces the release of inflammatory factors, thereby exacerbating tissue inflammatory responses and aging. Using ASOs targeting the IgG recycling receptor *Fcgrt* to reduce intracellular IgG levels in multiple tissues of aged mice, improved glucose tolerance and insulin sensitivity, and effectively mitigated the aging of multiple organs, providing new insights for aging intervention. Given the decline in immune system function in the elderly, inhibiting or blocking IgG may produce side effects with the risk of infection (Nandakumar & Holmdahl, 2008; Peter et al., 2020). Therefore, further research is needed to explore the dosage and intervention time window of gene therapy targeting immune system.

(4). **Gene therapy for rejuvenation of senescent cells**

The accumulation of senescent cells is a fundamental driver of organ and organismal aging. With exploration of mechanisms underneath cellular senescence, targeted gene therapy to rejuvenate senescent cell provides a novel direction for delaying aging.

Cell cycle arrest is one of the prominent characteristics of senescent cells. In aging intervention research, regulating the cell cycle to promote cellular rejuvenation is considered a potentially strategy (Wang et al., 2025). For example, Ye et al. used LV to deliver *Sirt2* to aged mouse hearts, counteracting senescence of cardiac cells and improving functional parameters such as ejection fraction by inhibiting the cyclin-dependent kinase inhibitor CDKN2B (Chen et al., 2024a; Ye et al., 2023). Additionally, Macedo et al. and Ribeiro et al. demonstrated that overexpression of only the N-terminally truncated form of *FOXM1* (*FOXM1*-dNdK) effectively promoted cell division in fibroblast from a patient with Hutchinson-Gilford Progeria Syndrome (HGPS) (Macedo et al., 2018). Furthermore, cyclically induced overexpression of *Foxm1* in HGPS mouse model successfully improved pathological aging phenotypes in multiple tissues including skin, adipose, aorta, and bone, and extended maximum lifespan by 28% (Ribeiro et al., 2022). However, despite this strategy showing enormous potential in promoting cell proliferation and regeneration, its potential tumorigenic risk cannot be overlooked, necessitating further in-depth exploration of long-term intervention safety to ensure its feasibility and reliability in clinical applications.

Yamanaka factors (OSKM), namely OCT4, SOX2, KLF4, and c-MYC, as reprogramming factors, play important roles in reshaping epigenetics and promoting rejuvenation of senescent cells (Narayan et al., 2017; Takahashi et al., 2007). Liu et al. reprogrammed HGPS fibroblasts to iPSCs, restoring aging-related nuclear membrane and epigenetic changes (Liu et al., 2011). Gill et al. developed a maturation phase transient reprogramming strategy, removing OSKM factors when reprogramming enters the maturation phase (around 13 days). This strategy reversed the epigenetic state of senescent cells, and restored fibroblasts' capability to secrete collagen and migrate (Gill et al., 2022). Using AAV9 or transgenic mouse models to periodically induce OSKM expression was proven to activate tissue-resident stem cell activity, alleviate cellular senescence, and demonstrate potential in delaying organ degeneration, promoting tissue regeneration, and extending lifespan, including increased thickness of epidermis and dermis layers in mouse skin, reduced splenic degeneration, decreased renal tubular damage levels, thickened gastric epithelium, restored visual function, and improved muscle regeneration capacity (Alle et al., 2022; Macip et al., 2024; Ocampo et al., 2016; Parras et al., 2023; Roux et al., 2021). For instance, Wang et al. found that inducing myofiber-specific OSKM expression enhances muscle stem cell activity, thereby promoting muscle regeneration (Wang, Rabadan Ros, et al., 2021).

However, this strategy also presents certain risks and challenges. First, OSKM expression may erase the terminal differentiation state of somatic cells, thereby altering cell "identity", potentially leading to loss or abnormality of cell function. Second, continuous reprogramming increases the risk of teratoma occurrence (Abad et al., 2013; Olova et al., 2019). Therefore, the specific mechanisms and roles of reprogramming factors in delaying aging require further investigation. Simultaneously, there is a need to discover new cellular rejuvenation factors that promote cellular regeneration without affecting cell "identity". Jing et al. used a CRISPR activation screening system to identify a series of pro-rejuvenation factors, among which the transcription factor SOX5 restores cellular vitality by activating HMGB2 expression (Jing et al., 2023). Delivering SOX5 using LV can delay mouse articular chondrocyte senescence, promote articular cartilage regeneration, and improve osteoarthritis symptoms. Importantly, SOX5 gene therapy did not cause changes in cell identity, suggesting better safety and efficacy for long-term intervention.

## 4 Clinical translation of gene therapy for aging: challenges and opportunities

Gene therapy represents a promising frontier in aging intervention, with a couple of clinical applications progressing through investigational stages. TERT gene therapy exemplifies this potential, having demonstrated improvements in aging-related molecular biomarkers and extended lifespan in murine models (Jaijyan et al., 2022). This preclinical success has translated into human trials, including a Phase I study (NCT04133649) evaluating an AAV carrying human TERT (AAV-hTERT) for the treatment of aging and age-related disorders. Notably, this trial prioritizes safety assessment, investigating the tolerability of intravenously administered AAV-hTERT, a critical step in establishing the therapeutic window for gene therapy approaches. The emphasis on comprehensive safety evaluation reflects the field's recognition that successful translation of gene therapy for aging intervention requires rigorous demonstration of safety profiles.

Genomic instability represents a paramount safety concern in gene therapy, particularly with integrating vectors such as LV that can disrupt essential genes through random integration into the human genome. This risk was starkly demonstrated in early clinical trials for severe combined immunodeficiencies (SCID), where insertional mutagenesis induced T-cell leukemia in several patients (Fischer & Hacein-Bey-Abina, 2020; Hacein-Bey-Abina et al., 2008). These early setbacks highlighted additional safety challenges that continue to shape the field. Furthermore, tumorigenicity remains a significant concern, particularly in OSKM-mediated cellular reprogramming protocols. These transcription factors may induce aberrant activation of proto-oncogenes. For example, c-Myc concurrently compromises tumor suppressor networks through mechanisms including inactivation of the p53 signaling pathway (Marión et al., 2009; Ohnishi et al., 2014). This process of reversing cell differentiation can confer a proliferative advantage upon certain reprogrammed cells. While technological advances, including high-fidelity Cas9 variants, have substantially improved targeting specificity, achieving clinical-grade precision remains an ongoing pursuit. Consequently, long-term surveillance is mandatory for all gene therapy applications, and continued optimization of these technologies is essential for safe clinical translation.

Immune-mediated complications represent a critical barrier to the clinical application of gene therapy for aging intervention. These adverse reactions, including hepatotoxicity and immune-mediated rejection of therapeutic vectors, have emerged as significant safety concerns in clinical trials (Chand et al., 2021). Despite prophylactic immunomodulation strategies, effectively managing vector-induced immune responses remains a substantial challenge (Strzelec et al., 2023). Notably, achieving the systemic distribution necessary for comprehensive aging intervention often requires elevated vector doses, substantially increasing toxicity risks (Ertl, 2023, Jagadisan & Dhawan, 2023). This creates a critical therapeutic dilemma: balancing efficacy with safety. Successful clinical translation of gene therapy for aging intervention requires integrated solutions addressing these interconnected challenges. Priority areas include: (1) Developing refined delivery approaches with improved safety profiles; (2) Establishing standardized immunomodulation protocols; (3) Optimizing vector design for enhanced tissue specificity or systematic application; (4) Conducting rigorously designed clinical trials to validate safety and efficacy.

Organ aging exhibits remarkable heterogeneity and asynchrony, driven by tissue-specific vulnerabilities and distinct microenvironmental factors (Nie et al., 2022, Tian et al., 2023). Each organ system displays characteristic aging signatures: the nervous system undergoes functional decline through synaptic plasticity deficits, while intestinal epithelia experiences diminished regenerative capacity secondary to stem cell exhaustion (Yun, 2015; Wilson et al., 2023). Sex differences and environmental stressors further amplify this organ-specific divergence, revealing aging as simultaneously a unified systemic process and a compartmentalized phenomenon (Hägg & Jylhävä, 2021). This dual nature presents significant challenges for gene therapy development in aging intervention. System-wide approaches targeting universal pathways, exemplified by telomerase activation via hTERT, encounter substantial delivery barriers. However, this complexity also creates therapeutic opportunities: combining systemic modulators such as senolytics with targeted gene therapy offers potential synergistic effects across multiple organ systems. Recent advances in multi-omics aging clock technologies now enable precise stratification of aging populations, supporting the development of personalized therapeutic regimens (Wang etal., 2023; Zheng et al., 2024; Li et al., 2023a). These approaches effectively balance broad-spectrum interventions with precision targeting, representing a paradigm shift in gerotherapeutic strategy.

The ethical regulation represents a critical consideration in translating gene therapy for aging intervention. Unlike monogenic disorders with clear genetic targets, the complexity of aging necessitates the development of comprehensive ethical guidelines and regulatory frameworks to ensure clinical translation (Aging Biomarker et al., 2024; Aging Biomarker et al., 2025; Peng et al., 2024; Li et al., 2023a). Access and affordability present additional challenges. Current gene therapy manufacturing limitations and high production costs create significant barriers to widespread implementation. Developing strategies to enhance manufacturing scalability and reduce costs will be essential for ensuring equitable access to these interventions.

The ongoing NCT04133649 trial evaluating AAV-hTERT marks an important milestone in assessing safety and efficacy for aging-related conditions. While this trial employs intravenous administration, future investigations may explore alternative delivery routes to minimize immune responses and improve targeted delivery (Zhu et al., 2024). Moreover, merging technologies offer promising solutions to current limitations. Novel vector designs incorporating "humanized" components demonstrate reduced immunogenicity. Additionally, innovative immunomodulatory strategies, including nanoparticle-based delivery approaches and B cell depletion protocols, show potential for overcoming existing barriers (Earley, Piletska, Ronzitti, & Piletsky, 2023, Kastner et al., 2025, Singh, Pandey, Malemnganba, & Prajapati, 2024). These advances collectively pave the way for more effective and accessible gene therapy approaches in aging medicine.

## 5 Conclusions and future perspectives

Gene therapy has emerged as a promising avenue for aging intervention, offering precise tools to target and modify key molecular pathways involved in aging-related decline. The advancements in gene editing technologies, such as base editing, and prime editing, have significantly improved the precision and efficiency of genetic modifications. In addition to traditional gene editing technologies like CRISPR-Cas systems, RNA-editing methods are emerging as powerful tools for precise genetic modifications, such as bridge RNA systems (Cheng et al., 2024; Durrant et al., 2024). This technology could revolutionize gene therapy by enabling more precise and efficient genetic modifications, particularly in the context of aging and aging-related diseases. Furthermore, recent advancements in artificial intelligence (AI) and computational tools, such as AI-driven imaging and AlphaFold, are revolutionizing personalized aging therapies by improving early diagnosis, predicting treatment responses, and accelerating drug discovery (Su et al., 2024; Li et al., 2023a; Sun et al., 2023; Zhao et al., 2025; Zheng et al., 2024; Wang et al., 2024c). These innovations hold great promise for developing targeted interventions that extend lifespan and improve healthspan.

Future research should also focus on integrating gene therapy with other rejuvenation strategies, such as stem cell therapies, small molecule interventions, and metabolic reprogramming (Yi et al., 2012; Lei et al., 2025). Additionally, large-scale CRISPR screening approaches and multi-omics analyses could further elucidate novel aging-related targets, paving the way for next-generation genetic interventions. High-throughput gene screening, made possible by sgRNA libraries, has become a powerful tool for decoding aging regulatory networks (Jia-Xin et al., 2024; Liu et al., 2018; Wei et al., 2021; Yao et al., 2024). For example, genome-wide knockout and activation screenings in human mesenchymal stem cells have identified novel aging-related genes such as *KAT7* and *SOX5*, advancing our understanding of aging mechanisms (Jing et al., 2023; Wang, Zheng, et al., 2021). Despite these advances, genome-wide CRISPR screening has limitations, such as masking genes truly linked to target traits and increasing false-negative rates. This underscores the importance of screening genes in specific biological pathways for deeper insights into aging. Studies have shown that RPL22 drives cellular senescence by disrupting rDNA heterochromatin stability, while histone variant H2AZ1 and nuclear transport protein XPO7 play roles in human stem cell senescence (Li et al., 2023b; Li et al., 2024a; Li et al., 2025b). The heterogeneity of aging across organs also poses challenges in identifying regulatory factors that restore cell vitality and tissue function in aged individuals. *In vivo* screening platforms, like the one established by Ruetz et al., offer promising approaches. They identified that knocking out *Slc2a4* enhances neural stem cell regeneration in aged mice (Ruetz et al., 2024). As *in vivo* and single-cell screening technologies evolve, they will likely reveal more about aging mechanisms and guide gene therapy strategies (Cheng et al., 2023; Liscovitch-Brauer et al., 2021; Uijttewaal et al., 2024).

**Fig. 4:**
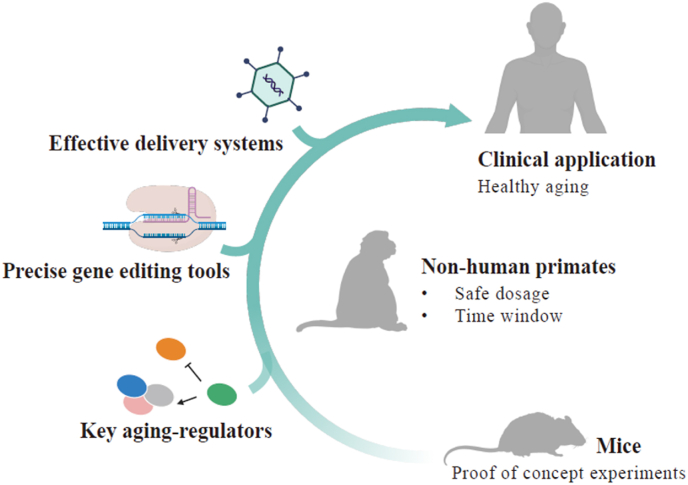
**Perspective of gene therapy for aging intervention.** The diagram illustrates the concept of using gene therapy to intervene in aging. As a proof-of-concept, gene therapy has been shown to alleviate aging and extend lifespan in mice. Both the identification of more aging regulator and the development of gene editing approaches will advance the gene therapy for aging intervention. Gene therapy in non-human primates is crucial for exploring dosage and intervention time windows. These steps establish a foundation for future clinical applications aimed at achieving healthy human aging.

The future of gene therapy and aging intervention is promising (Fig. 4). Optimizing gene-editing tools can boost precision and efficiency, while innovative delivery systems promise to enhance vector capacity and targeting specificity, addressing the challenges of multi-gene targeting. Moreover, the combined application of gene therapy with stem cell therapy, small molecule drug interventions, and lifestyle adjustments is expected to form a comprehensive treatment system that delays aging from multiple perspectives, creating personalized treatment plans based on individual aging characteristics and genetic backgrounds.

## CRediT authorship contribution statement

**Yaobin Jing:** Writing – original draft, Conceptualization. **Jie Ren:** Writing – review & editing. **Jing Qu:** Writing – review & editing, Investigation, Funding acquisition, Conceptualization. **Guang-Hui Liu:** Writing – review & editing, Investigation, Funding acquisition, Conceptualization.

## Declaration of competing interest

The authors declare no conflict of interest.
